# Morning cortisol as an indicator of arterial stiffness in patients with type 2 diabetes: prospective cohort study and Mendelian randomization study

**DOI:** 10.3389/fendo.2025.1687909

**Published:** 2025-11-25

**Authors:** Zhuomeng Hu, Lin Sun, Ying Peng, Juan Shi, Yihua Guo, Qianhua Fang, Cong Liu, Xing Wei, Jie Hong, Weiqiong Gu, Weiwei Zhou, Weiqing Wang, Yifei Zhang

**Affiliations:** 1 Department of Endocrine and Metabolic Diseases, Shanghai Institute of Endocrine and Metabolic Diseases, Ruijin Hospital, Shanghai Jiao Tong University School of Medicine, Shanghai, China; 2 Shanghai National Clinical Research Center for Metabolic Diseases, Key Laboratory for Endocrine and Metabolic Diseases of the National Health Commission of the PR China, Shanghai Key Laboratory for Endocrine Tumor, State Key Laboratory of Medical Genomics, Ruijin Hospital, Shanghai Jiao Tong University School of Medicine, Shanghai, China

**Keywords:** cohort study, Mendelian randomization, cortisol, arterial stiffness, coronary atherosclerosis, type 2 diabetes

## Abstract

**Introduction:**

Elevated cortisol levels have been linked to arterial stiffness, but the evidence for this association remains controversial. We aimed to elucidate this relationship and to explore potential mediation pathways.

**Methods:**

To investigate the relationship between morning cortisol and arterial stiffness, two approaches were employed. First, we used linear mixed-effects (LME) models and mediation analysis in a prospective cohort study (n=1,235; average follow-up of 3.5 years) in type 2 diabetes (T2D), featuring repeated brachial-ankle pulse wave velocity (baPWV) measurements (2–8 per participant; 4,143 total) to assess arterial stiffness. Second, a two-step Mendelian randomization (MR) study was conducted using summary data of genome-wide association studies (GWAS) of CORtisol NETwork (CORNET) and UK Biobank (UKB). Arterial stiffness was measured by baPWV in the cohort study, with coronary atherosclerosis from UKB serving as the validation outcome.

**Results:**

The prospective study included participants with a mean age of 54.3 ± 11.3 years (65.3% male) and a mean baseline baPWV of 16.06 ± 3.23 m/s. It revealed that each 1-unit increase in log_10_Cortisol was associated with a 0.67 m/s (95% CI: 0.25–1.10, P = 0.002) increase in baPWV. Mediation analysis indicated that systolic blood pressure (SBP), diastolic blood pressure (DBP), and mean arterial pressure (MAP) all partially mediated the association between morning cortisol and arterial stiffness, with SBP contributing the largest proportion (18.68%, 95% CI: 16.48–23.66%; P = 0.033). The two-step MR analysis further supported that SBP could mediate the positive relationship between morning cortisol and coronary atherosclerosis.

**Conclusions:**

This research provides both observational and genetic evidence indicating a potential causal relationship between morning cortisol and arterial stiffness, with SBP as a key mediator.

## 1 Introduction

Cardiovascular diseases (CVD), the foremost contributor to morbidity and mortality, are characterized by complex structural and functional alterations taking place in the arterial system and are significantly influenced by stress, which can trigger disease in individuals with high atherosclerotic plaque burden (1–3). Arterial stiffness is widely accepted as an early indicator of coronary atherosclerosis and independently predicts cardiovascular mortality (4).

Cortisol, the final product produced by the hypothalamic-pituitary-adrenal (HPA) axis, has been recognized as a core hormonal mediator of chronic stress (5–7). Several cross-sectional studies have demonstrated a significant correlation between elevated cortisol concentrations and major CVD risk factors, including hypertension, triglycerides (TG), fibrinogen, and leptin (8–10). Elevated cortisol levels may also induce glucocorticoid receptor resistance, increasing susceptibility to inflammatory-related diseases and potentially accelerating arterial stiffness (11). However, the relationship between cortisol and arterial stiffness or CVDs remains inconclusive (12–16), with most existing research limited by retrospective or crossover study design, relatively small sample sizes and scarcity of baPWV measurements (≤ 2).

In this study, we aimed to clarify the association between morning serum cortisol levels and arterial stiffness and to identify potential mediators in a repeated-measures prospective cohort study involving Asian populations. As an analytical approach for causal inference, Mendelian randomization (MR) offers methodological advantages by reducing susceptibility to residual confounding (17, 18). Leveraging this method, we employed a two-step MR approach utilizing European population genetic data, which enabled examination of causal relationships and mediation pathways.

## 2 Materials and methods

### 2.1 Prospective cohort study design

#### 2.1.1 Study population

In this prospective cohort study, subjects were enrolled from the National Metabolic Management Center (MMC) at Ruijin Hospital, Shanghai Jiao Tong University School of Medicine. The MMC, recognized as a standard diabetes care system in China, is detailed in previous publications (19–24), with its protocol accessible under ClinicalTrials.gov identifier: NCT03811470. Briefly, the MMC is an Internet-based health information platform for managing diabetes and other metabolic diseases.

**Figure 1:**
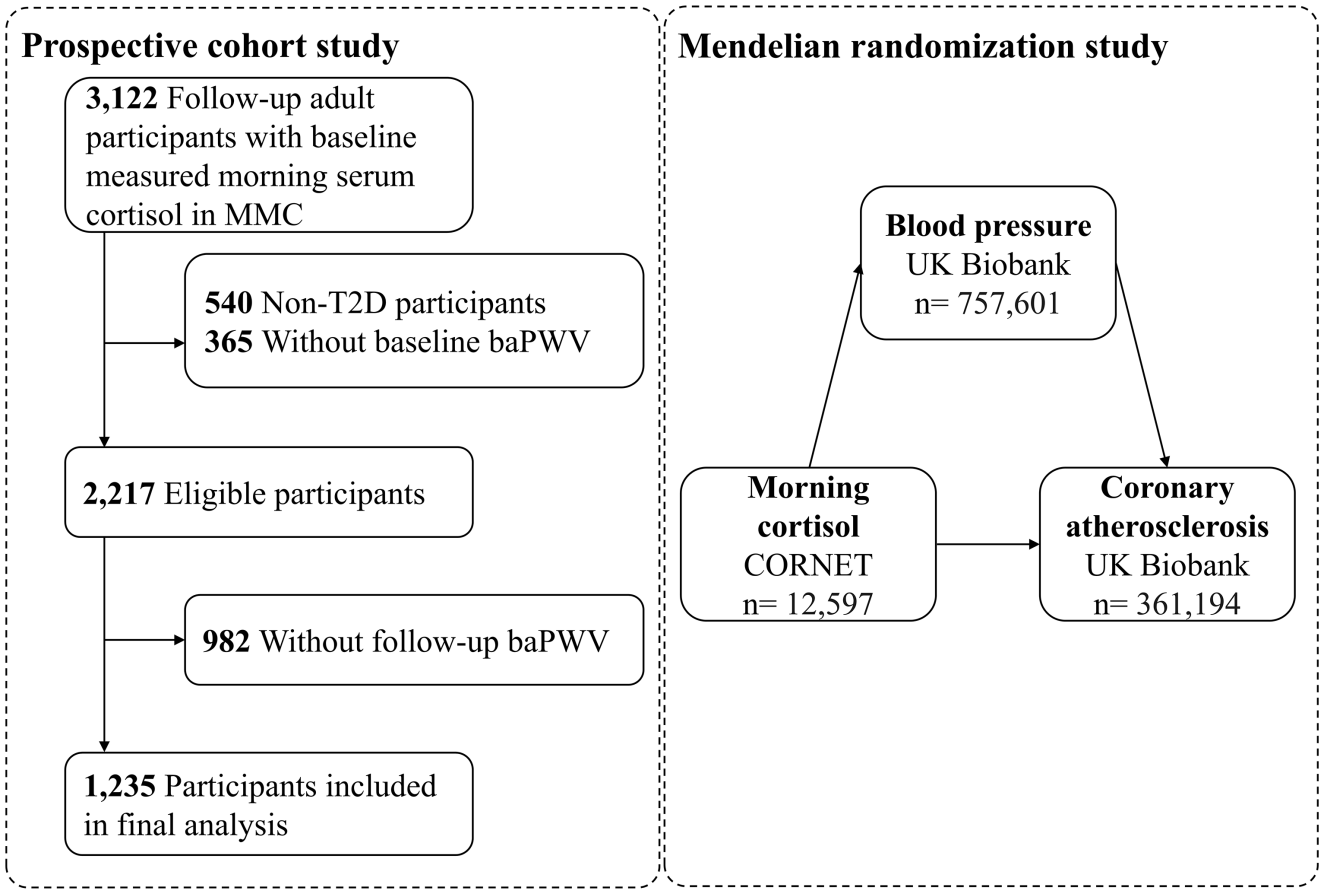
Study flowchart. MMC, Metabolic Management Center; T2D, type 2 diabetes; baPWV, brachial-ankle pulse wave velocity.

A total of 3,122 participants aged 18 years or older were followed in MMC from June 2017 to June 2024, all of whom had baseline morning serum cortisol measured and no history of drug abuse, hormonal medications, pituitary or adrenal diseases, related surgeries, or radiotherapy. Initially, we excluded participants who did not have type 2 diabetes (T2D) (n=540). Further exclusions were made due to the absence of baseline arterial stiffness measurements (n=365) or the absence of follow-up arterial stiffness measurements (n=982), and finally 1,235 subjects were incorporated into the main analysis (**Figure 1**). Arterial stiffness was assessed using all available baPWV data, including both initial baseline measurements and subsequent follow-up assessments.

#### 2.1.2 Anthropometric and laboratory measurements

As described in the protocol of MMC, during both the registration and follow-up visits, a standardized digital medical record system was used to collect information. Recorded variables included basic demographics (e.g., age, sex); history of previous CVD (including stroke, heart failure, or coronary heart disease); smoking; drinking (recorded as ‘yes’ for participants who drank weekly or almost weekly); sleep duration; and the use of antihypertensive agents and lipid-lowering agents. Waist circumference was assessed at the midpoint between the lower border of the costal arch and the upper border of the iliac crest. Systolic blood pressure (SBP) and diastolic blood pressure (DBP) were recorded with the subject in the sitting position after ≥ 5 minutes of rest. Mean arterial pressure (MAP) was calculated as (1/3)*SBP+(2/3)*DBP (25). Visceral fat area (VFA) and subcutaneous fat area (SFA) were conducted using bioelectrical impedance analysis (DUALSCAN, HDS-2000; Omron Healthcare Co., Ltd., Kyoto, Japan) (26).

After overnight fasting, blood samples were collected in the morning and included serum cortisol, glycated hemoglobin A1c (HbA1c), fasting blood glucose (FBG), total cholesterol (TC), TG, high-density lipoprotein cholesterol (HDL-C), low-density lipoprotein cholesterol (LDL-C) and other laboratory parameters. Morning serum cortisol was measured by using an Access Immunoassay System (Beckman Coulter Inc., Fullerton, CA, USA), with a normal range of 6.7–22.6 µg/dl.

Arterial stiffness was measured by baPWV in the cohort study. As described in previous studies (22, 27), baPWV was measured by an automated recording apparatus (BP-203RPE III, form PWV/ABI, Omron Healthcare Co.) in the supine position after resting for ≥ 5 minutes. Simultaneous readings were taken from the brachial and tibial arteries. The study measured both the pulse wave transit time—the interval between the detection of pulse waves at the brachial and tibial arteries—and the transit distance, measured from the upper arm to the ankle. The baPWV was derived as the ratio of transmission distance per the transit time. The average of the right and left baPWV was used for analysis. All participants, if possible, were assessed for baPWV at least three times over a five-year period by an independent, trained observer: at baseline, either year 2 or 3, and year 5. Individuals with a minimum of two baPWV assessments throughout the follow-up period were included in the analysis, and the number of baPWV measurements for each participant ranged from 2 to 8, with a total of 4,143 measurements.

#### 2.1.3 Cohort statistical analysis

Group comparisons were conducted using the t-tests (or ANOVA when appropriate) for continuous variables, while categorical differences were evaluated through χ^2^ tests. Linear mixed-effects (LME) models were utilized to investigate the relationship between baseline morning cortisol and repeated measurements of baPWV, with random effects specified for individuals, and utilized a penalized spline method to fit a smoothing curve. This relationship was further examined in stratified quartiles of morning cortisol levels. Morning serum cortisol was log10-transformed (log_10_) before statistical analysis. Two models, adjusted for key covariables, were constructed: Model 1, which was adjusted for age, sex (female or male), and MAP; and Model 2, which was additionally adjusted for all of the variables in Model 1 plus diabetes duration, VFA, LDL-C, HbA1c, smoking status (current, former and quit ≤ 12 months, never or quit > 12 months), drinking status (yes or no), sleep duration (< 7 h, 7–9 h, > 9 h), history of cardiovascular disease (yes or no), antihypertensive agents (yes or no), and lipid-lowering agents (yes or no). P values were adjusted for multiple testing using the Benjamini–Hochberg false discovery rate (FDRB-H) method. All of the statistical analyses were conducted using R (version 4.3.0) with the “lme4” and “rms” packages. Sensitivity analyses were performed to assess the robustness of the model by sequentially removing one variable at a time from the main model, including VFA, LDL-C, HbA1c, or agents (including antihypertensive agents and lipid-lowering agents). Using restricted cubic spline (RCS) analysis based on Model 2, we examined the nonlinear association between morning cortisol and baPWV.

We further performed mediation analysis to evaluate the effects of mediators on the relationship between morning cortisol and baPWV. The potential mediators, available from the follow-up visits, comprised anthropometric indicators (SBP, DBP, MAP, BMI, waist circumference, hip circumference, VFA, and SFA), blood lipids (TC, TG, HDL-C, LDL-C), FBG and lifestyle factors (smoking, drinking, and sleep duration). Two separate LME models with random intercepts were fitted in the single-mediator model. In the first step, a model was designed to analyze the exposure-mediator association. Only if this association was significant did we proceed to the second step, which focused on the mediator-outcome association. The mediation or indirect effects were measured to determine the extent to which the influence of morning cortisol on arterial stiffness could be attributed to or was facilitated by these risk factors, thereby elucidating the pathway through which morning cortisol potentially influences arterial stiffness. The mediated proportion was calculated by computing the ratio of the indirect effect (β_1_ × β_2_) to the total effect (β_3_) of morning serum cortisol on arterial stiffness. Standard errors were derived using the bootstrap method, with effect estimates extracted from LME models (**Supplementary Figure S1**).

### 2.2 Two-step MR study design

#### 2.2.1 Genetic instruments of morning cortisol

Instrumental variables (IVs) for morning cortisol were selected from the CORtisol NETwork (CORNET) consortium genome-wide association study (GWAS) data (n=12,597), excluding individuals currently using glucocorticoids, pregnant or breastfeeding women, and twins (28). Cortisol measurements were obtained via immunoassay from morning blood samples (7:00-11:00 AM; mean concentration 17.3 µg/dL). In accordance with the recent MR research concerning cortisol (29–31), we identified three single-nucleotide polymorphisms (SNPs) that were independently associated (r² < 0.3) with morning cortisol levels at genome-wide significance (P < 5*10^-8^) (**Supplementary Table S1**). These included two *SERPINA6* variants (rs12589136, rs11621961) influencing corticosteroid-binding globulin and one *SERPINA1* variant (rs2749527) affecting α1-antitrypsin. The SNPs were adjusted for age, sex, and principal components of ancestry, accounting for 0.54% of the variation in morning cortisol levels (28). The F-statistics for IVs indicated that genetic variants significantly predicted morning cortisol levels (F-statistics > 10) (**Supplementary Table S1**) (31).

#### 2.2.2 Genetic associations of coronary atherosclerosis

Coronary atherosclerosis was used as the validation outcome in the MR study. External validation was performed using coronary atherosclerosis phenotypes (ukb-d-I9_CORATHER), with GWAS summary data sourced from the UK Biobank, which includes 361,194 participants (**Supplementary Table S2**, available on the IEU OpenGWAS project website at https://gwas.mrcieu.ac.uk/) .

#### 2.2.3 Genetic associations of intermediate phenotypes

We identified blood pressure (SBP and DBP) as potential mediators. The study utilized GWAS data from 757,601 UK Biobank participants of European descent (**Supplementary Table S2**, accessible at https://gwas.mrcieu.ac.uk/). When considering blood pressure (SBP and DBP) as exposures, we employed a P value threshold of 5*10^-8^, and variants were grouped based on a linkage disequilibrium r² threshold of 0.3 to exclude those exhibiting high correlations.

Ethical approval for all studies was obtained from the relevant institutional or national review boards, with participants providing written informed consent.

#### 2.2.4 MR statistical analysis

We performed two-sample MR analysis to explore the potential causal relationships between morning cortisol levels and coronary atherosclerosis. The primary method was inverse-variance weighted (IVW).

In addition, two-step MR analysis was used to examine the mediation effect of blood pressure (SBP and DBP) on the relationship between morning cortisol and coronary atherosclerosis. In the initial phase of the two-step MR, we assessed the effect of morning cortisol (exposure) on blood pressure (outcome) (**Supplementary Figure S2A**). The blood pressures indicating MR evidence in step one were subsequently examined in step two, in which the effect of blood pressure (exposure) on coronary atherosclerosis (outcome) was estimated (**Supplementary Figure S2B**). For sensitivity analyses, the potential pleiotropic effects were assessed by the MR-Egger intercept, and Cochran’s Q statistical analysis was used to evaluate possible heterogeneity. We reported the effects of per unit change in gene expression level on exposures as β or odds ratios (ORs) with 95% CIs. The MR analysis was all performed utilizing the “TwoSampleMR” and “MendelianRandomization” packages in R (version 4.3.0).

## 3 Results

### 3.1 Prospective cohort study

#### 3.1.1 Clinical characteristics

The analysis included 1,235 patients with T2D followed for an average of 3.5 (SD, 1.9) years. Demographic and metabolic features of patients by sex are detailed in **Table 1**. The cohort had a mean age of 54.3 (SD, 11.3) years, with 65.3% being male. The baseline morning serum cortisol concentration was 12.31 (IQR, 9.53 to 14.74) μg/dl, with higher levels observed in male participants. The mean baPWV for the cohort was 16.06 (SD, 3.23) m/s at the first visit, increasing to 17.24 (SD, 3.65) m/s at the last follow-up visit.

**Table 1:** Baseline characteristics of all participants.

Characteristics	Total (n=1,235)	Male (n=807)	Female (n=428)	P value
Age (years)	54.3 ± 11.3	53.4 ± 11.2	56.0 ± 11.4	<0.001
Diabetes duration (months)	106.81 ± 93.17	108.56 ± 93.29	103.50 ± 92.98	0.379
Follow-up time (y)	3.5 ± 1.9	3.4 ± 1.9	3.6 ± 2.0	0.017
Drinking, n (%)	146 (11.91%)	141 (17.65%)	5 (1.17%)	<0.001
Smoking status, n (%):				<0.001
Current	289 (23.75%)	283 (35.73%)	6 (1.41%)	
Former, quit≤ 12 mo	43 (3.53%)	43 (5.43%)	0 (0.0%)	
Never or quit> 12 mo	885 (72.72%)	466 (58.84%)	419 (98.59%)	
Sleep duration, n (%):				0.123
< 7 h	174 (16.42%)	109 (15.98%)	65 (17.20%)	
7–9 h	748 (70.57%)	494 (72.43%)	254 (67.20%)	
> 9 h	55 (13.02%)	79 (11.58%)	59 (15.61%)	
History of CVD (%)	192 (15.62%)	116 (14.46%)	76 (17.80%)	0.147
Waist circumference (cm)	93.7 ± 10.3	95.5 ± 9.4	90.4 ± 11.0	<0.001
BMI (kg/m^2^)	26.19 ± 4.12	26.33 ± 3.84	25.93 ± 4.60	0.128
SBP (mmHg)	130.1 ± 18.0	129.8 ± 17.5	130.7 ± 19.0	0.369
DBP (mmHg)	76.1 ± 11.2	77.5 ± 10.7	73.5 ± 11.8	<0.001
MAP (mmHg)	94.1 ± 12.2	94.9 ± 11.9	92.6 ± 12.7	0.001
VFA (cm^2^)	108.57 ± 40.80	112.47 ± 39.88	100.98 ± 41.53	<0.001
SFA (cm^2^)	193.61 ± 67.97	191.66 ± 62.77	197.40 ± 77.03	0.209
Morning serum cortisol (μg/dl)	12.31 ± 3.79	12.50 ± 3.77	11.96 ± 3.80	0.016
TC (mmol/L)	4.04 ± 0.75	3.99 ± 0.71	4.14 ± 0.83	0.015
TG (mmol/L)	2.04 ± 1.96	2.11 ± 1.95	1.92 ± 1.99	0.104
HDL-C (mmol/L)	1.18 ± 0.31	1.11 ± 0.27	1.33 ± 0.33	<0.001
LDL-C (mmol/L)	2.89 ± 0.94	2.84 ± 0.91	2.98 ± 0.98	0.013
HbA1c (%)	7.86 ± 1.77	7.94 ± 1.83	7.70 ± 1.65	0.020
FBG (mmol/L)	8.52 ± 3.05	8.56 ± 2.92	8.46 ± 3.28	0.581
First baPWV (m/s)	16.06 ± 3.23	15.97 ± 3.18	16.23 ± 3.33	0.187
Last baPWV (m/s)	17.24 ± 3.65	17.06 ± 3.52	17.60 ± 3.89	0.017
Lipid-lowering agents, n (%)	311 (25.43%)	195 (24.50%)	116 (27.17%)	0.341
Antihypertensive agents, n (%)	515 (42.14%)	347 (43.65%)	168 (39.34%)	0.164

Values are mean ± SD or number (percentage).

CVD, cardiovascular disease; BMI, body mass index; SBP, systolic blood pressure; DBP, diastolic blood pressure; MAP, mean arterial pressure; VFA, visceral fat area; SFA, subcutaneous fat area; TC, total cholesterol; TG, triglycerides; HDL-C, high-density lipoprotein cholesterol; LDL-C, low-density lipoprotein cholesterol; HbA1c, glycated hemoglobin A1c; FBG, fasting blood glucose; baPWV, brachial-ankle pulse wave velocity.

#### 3.1.2 Dose-response relationship between morning cortisol and baPWV

**Figure 2:**
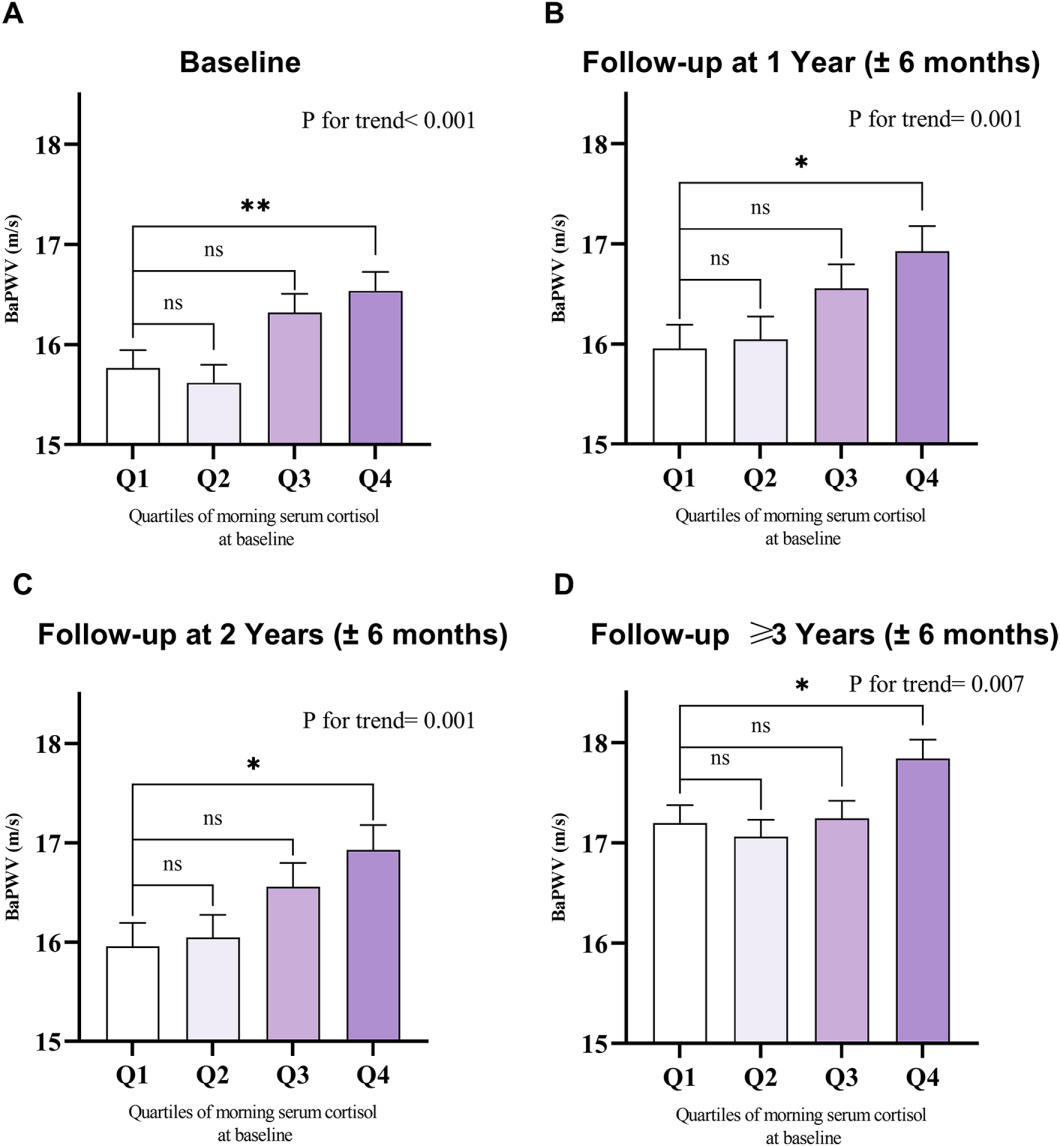
Mean with SEM of baseline and follow-up baPWV among the baseline morning serum cortisol quartiles. Value of baPWV at **(A)** baseline (n=1,239), **(B)** 1-year follow-up (n=796), **(C)** 2-year follow-up (n=606), and **(D)** follow-up ≥3 years (n=902), grouped by quartiles of baseline morning serum cortisol levels. SEM, standard error of the mean; baPWV, brachial-ankle pulse wave velocity. Comparisons were performed using one-way ANOVA. Quartiles of morning serum cortisol at baseline: Q1: 5.00-9.52 μg/dl; Q2: 9.53-11.89 μg/dl; Q3: 11.90-14.73 μg/dl; Q4: 14.74-22.50 μg/dl. The higher the morning serum cortisol level, the higher the follow-up baPWV value observed at multiple follow-up time points. *P < 0.05, **P < 0.01, ***P < 0.001, ns, not significant.

To minimize bias arising from the unequal number of patients across groups when stratified by baseline cortisol quartiles (Q1 to Q4), we accounted for follow-up times in the one-way ANOVA. This revealed that baPWV increased with higher cortisol levels at multiple follow-up time points (including baseline, 1 year, 2 years, and ≥3 years) (**Figure 2**). For each 1-unit increase in log_10_Cortisol, baPWV was observed to increase by 0.67 m/s (95% CI: 0.25–1.10, P = 0.002) in multivariate Model 2, adjusted for age, sex, MAP, diabetes duration, VFA, LDL-C, HbA1c, smoking status, drinking status, history of CVD, antihypertensive agents, and lipid-lowering agents. When baseline cortisol levels were categorized into quartiles, a gradual increase in the regression coefficients was observed (P for trend < 0.05 for all models), suggesting a dose-response relationship between cortisol quartiles and baPWV. Additionally, compared with lower morning cortisol (Q1 and Q2), higher morning cortisol (Q3 and Q4) correlated with a significant increase in baPWV of 0.75 m/s (95% CI: 0.48–1.01, P < 0.001) in the crude model and 0.46 m/s (95% CI: 0.20–0.72, P = 0.001) after full adjustments (Model 2) (**Table 2**). No significant interaction effects were observed between morning serum cortisol and any covariables (P > 0.05 for all interactions). In the RCS linear test based on Model 2, baseline morning serum cortisol was positively associated with follow-up baPWV (P for nonlinear=0.518, **Supplementary Figure S3**).

**Table 2:** Association between baseline morning serum cortisol and arterial stiffness progression.

Cortisol	Crude		Model 1		Model 2	
	β (95% CI)	P	β (95% CI)	P	β (95% CI)	P
Continuous						
Per 1 unit log_10_Cortisol increase	1.23 (0.79, 1.67)	<0.001	0.64 (0.26, 1.03)	0.001	0.67 (0.25, 1.10)	0.002
Quartiles (min-max)						
Q1 (5.00– 9.52)	Ref	Ref	Ref	Ref	Ref	Ref
Q2 (9.53– 11.89)	-0.02(-0.38, 0.35)	0.926	-0.08 (-0.40, 0.25)	0.650	0.02 (-0.35, 0.38)	0.927
Q3 (11.90– 14.73)	0.61 (0.24, 0.98)	0.001	0.36 (0.03, 0.69)	0.032	0.40 (0.03, 0.76)	0.032
Q4 (14.74– 22.50)	0.89 (0.50, 1.28)	<0.001	0.46 (0.12, 0.80)	0.008	0.55 (0.17, 0.92)	0.005
P for trend		<0.001		0.001		0.001
Categories						
Q1, Q2	Ref	Ref	Ref	Ref	Ref	Ref
Q3, Q4	0.75 (0.48, 1.01)	<0.001	0.45 (0.21, 0.68)	<0.001	0.46 (0.20, 0.72)	0.001

β coefficients were estimated using the linear mixed-effects (LME) models. P values were derived from the Restricted maximum likelihood. Model 1: adjusted for age, sex, and MAP. Model 2: all of the variables in Model 1 plus diabetes duration, VFA, LDL-C, HbA1c, smoking status, drinking status, history of CVD, antihypertensive agents, and lipid-lowering agents. CI, confidence interval; Log_10_, log-transformed with base 10; MAP, mean arterial pressure; VFA, visceral fat area; LDL-C, low-density lipoprotein cholesterol; HbA1c, glycated hemoglobin A1c; CVD, cardiovascular disease.

#### 3.1.3 Mediation analysis on multiple risk factors of arterial stiffness

**Figure 3:**
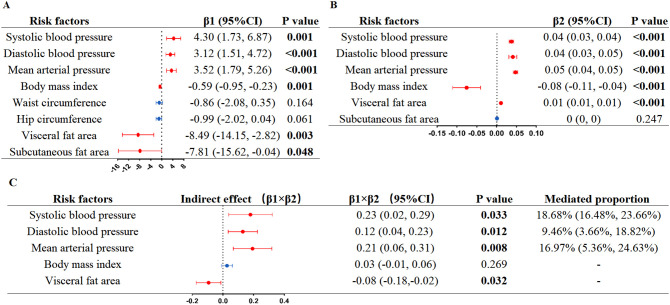
Linear mixed-effects models mediation analysis of morning serum log_10_Cortisol on arterial stiffness via risk factors. **(A)** Linear mixed-effects models analysis of morning serum log_10_Cortisol on risk factors; **(B)** Linear mixed-effects models analysis of risk factors on arterial stiffness; **(C)** Estimates for the effect of morning serum log_10_Cortisol on arterial stiffness explained by risk factors. β1, the effect of morning serum log_10_Cortisol on risk factor. β2, the effect of risk factors on arterial stiffness. P values were calculated from the restricted maximum likelihood. Log10, log-transformed with base 10.

We performed mediation analysis on multiple risk factors of arterial stiffness to explore the mediating effect. In a first step, based on the results of LME model analysis, six anthropometric risk factors (SBP, DBP, MAP, BMI, VFA, and SFA) were significantly associated with the morning serum log_10_Cortisol (**Figure 3A**). No significant results were observed for blood lipids (including TC, TG, HDL-C, LDL-C), FBG, or lifestyle factors (smoking, drinking, or sleep duration) (**Supplementary Table S3**). In a second step, we further evaluated the effect of the six risk factors mentioned previously on baPWV. The results demonstrated significant positive correlations between baPWV and four risk factors (SBP, DBP, MAP, and VFA), whereas there was a negative relationship between BMI and baPWV (β: -0.08, 95% CI: -0.11 to -0.04, P<0.001) (**Figure 3B**). Mediation analysis indicated that SBP mediated 18.68% (95% CI: 16.48–23.66, P = 0.033) of the effect of morning serum log_10_Cortisol on arterial stiffness, MAP mediated 16.97% (95% CI: 5.36–24.63, P = 0.008), and DBP mediated 9.46% (95% CI: 3.66–18.82, P = 0.012) (**Figure 3C**).

#### 3.1.4 Sensitivity analyses

Sensitivity analyses, performed by sequentially removing one variable at a time (including VFA, LDL-C, HbA1c, and agents) from the main model, confirmed the robustness of our results (**Supplementary Tables S4**, **S5**).

### 3.2 Two-step MR study

#### 3.2.1 Causal effects of morning cortisol on coronary atherosclerosis

MR analysis indicated that elevated morning cortisol levels were genetically associated with an increased risk of coronary atherosclerosis (IVW method: OR = 1.01, 95% CI: 1.00–1.01, P = 0.049) (**Table 3**).

**Table 3:** Two-step Mendelian randomization analysis of morning cortisol and coronary atherosclerosis via blood pressure.

Exposure	Total Effect		Direct Effect A		Direct Effect B	
	OR(95%CI)	P	β(95%CI)	P	OR(95%CI)	P
Systolic blood pressure	1.01 (1.00,1.01)	0.049	0.59 (0.16,1.01)	0.007	1.00 (1.00,1.00)	2.35E-138
Diastolic blood pressure			0.23 (-0.02,0.47)	0.069	1.00 (1.00,1.00)	2.34E-116

Total effect: indicates the effect of morning cortisol on coronary atherosclerosis; direct effect A: the effect of morning cortisol on blood pressure; direct effect B: the effect of blood pressure on coronary atherosclerosis. The total effect, direct effect A, and direct effect B were derived using the inverse-variance weighted method. All statistical tests were 2-sided. P < 0.05 was considered significant. OR, odds ratio; CI, confidence interval.

#### 3.2.2 Causal effects of morning cortisol on blood pressure

To address whether blood pressure played mediating roles in promoting coronary atherosclerosis, we first assessed the causal association between cortisol and blood pressure. Higher cortisol levels were significantly associated with increased SBP (IVW: β=0.59, 95% CI: 0.16–1.01, P = 0.007) (**Table 3**). However, no significant association was observed between cortisol and DBP (IVW method: β=0.23, 95% CI: -0.02 to 0.47, P = 0.069) (**Table 3**).

#### 3.2.3 Causal effects of blood pressure on coronary atherosclerosis

Next, we evaluated the causal effect of blood pressure on coronary atherosclerosis. The results revealed that elevated SBP was significantly associated with an increased risk of coronary atherosclerosis (IVW method: OR = 1.00, 95% CI: 1.00–1.00, P = 2.35E-138) (**Table 3**).

#### 3.2.4 Mediation effects of morning cortisol on coronary atherosclerosis through blood pressure

Two-step MR analysis demonstrated that SBP was a significant intermediate variable linking morning cortisol with coronary atherosclerosis. However, DBP showed no genetically predicted association with morning cortisol when assessed using the IVW method.

#### 3.2.5 Sensitivity analyses

Cochran’s Q test and MR-Egger intercept were performed to assess the robustness of the results (**Supplementary Table S6**). The MR-Egger intercept tests produced P-values > 0.05, suggesting that there is no horizontal pleiotropy in the analyses. Cochran’s Q test revealed there was no heterogeneity between cortisol and coronary atherosclerosis, as well as between cortisol and SBP, and between cortisol and DBP. However, when SBP and DBP were used as exposures, the results of Cochran’s Q test showed P<0.05, which indicated that there was heterogeneity among the SNPs in SBP and DBP. The impact on the results was minimal due to the use of the IVW method (32).

## 4 Discussion

In the study, findings from the observational study in Asian populations, along with results from the MR study in Europeans, demonstrated that there was a causal correlation between morning cortisol and arterial stiffness. In addition, SBP might serve as a mediator in this relationship.

Cortisol, a glucocorticoid hormone, is widely recognized as the “stress hormone” due to its pivotal role in the body’s stress response mechanism (6). Meanwhile, morning serum cortisol has seen extensive application across clinical research, spanning both observational and randomized studies (33–35). For decades, clinical research has consistently shown that exposure to psychological stressors, such as occupational pressure, marital discord, and economic instability, is associated with a higher risk of both CVD and diabetes (36–39). Gene function research evidence suggests that there is a correlation between chronic stress and epigenetic modifications of genes associated with glucocorticoids, potentially impacting the risk of CVD (40–43). However, the evidence linking cortisol levels to arterial stiffness and CVD events remains less conclusive, as most studies conducted so far have been cross-sectional or retrospective (12, 44–46). One prospective cohort study involving 271 Black and White children revealed that hair cortisol was positively correlated with CVD (13). Nevertheless, two studies in European populations showed that circulating plasma cortisol and waking cortisol were poor predictors of vascular disease or cardiovascular-related mortality (15, 16). The discrepancies in research conclusions may be due to methodological limitations, including insufficient participant numbers, cross-sectional study designs, short follow-up periods, and a limited number of baPWV measurements.

Here, we thus conducted a repeated-measures cohort study involving Asian populations, as well as a large-scale MR study involving European populations, to clarify this association. MR analysis, which examines the effects of lifelong exposure, is less susceptible to bias from confounding. Our findings, therefore, have provided both observational and genetic evidence of a causal relationship between morning cortisol levels and arterial stiffness, with SBP acting as a mediating factor. In addition, morning cortisol was positively associated with DBP in the cohort study but not significantly in the MR study (P = 0.07). This difference may be partly due to the different methods used in each study: MR studies use genetic variants, typically SNPs, which are linked to the exposures of interest but are less likely to be affected by these confounding factors. This makes MR studies better at providing a causal link, which could explain why the MR study showed no significant results for DBP compared with the cohort study (17). The underlying mechanisms are clearly intricate, necessitating additional research.

To date, there have been no current CVD prevention guidelines that consider morning cortisol as a predictor in risk assessment. Our results raise the possibility that the measurement of morning cortisol may serve as an accessible method for identifying adults who may be prone to arterial stiffness. We recommend setting a threshold above which preventive measures for arterial stiffness should be implemented in patients whose morning cortisol levels fall within the normal range. In addition, our findings indicate that cortisol may change blood pressure (especially SBP), which partially mediated the impacts of cortisol on arterial stiffness. This finding supports the American Heart Association’s current guidelines for intensive blood pressure lowering therapy (47), while also broadening our present knowledge of the mechanisms linking morning cortisol to arterial stiffness. Consistent with our findings, population studies have examined the correlation between SBP levels and variability and the risk of CVD across extended periods. The findings highlight that both time-averaged “antecedent” BP levels and “cumulative” BP measurements serve as robust indicators for forecasting the onset of major future cardiovascular events, including coronary heart disease, heart failure, stroke, and vascular dementia (48–51). Notably, our findings also demonstrated that higher morning cortisol concentrations were inversely related to VFA (β_1_ = –8.49, P = 0.003), whereas VFA showed a positive correlation with arterial stiffness (β_2_ = 0.01, P < 0.001) (**Figure 3**). These results were in line with previous reports (35, 52, 53) and suggest that VFA was represented as a masking effect. Additionally, we observed a negative association between BMI and baPWV (**Figure 3B**), which was consistent with some earlier studies (54–56). This seemingly contradictory phenomenon may be due to the fact that BMI, as a general indicator of obesity, does not distinguish differences in fat distribution, combined with the confounding effect of age in our population, where BMI decreased with age—a key driver of increased baPWV.

Our study has several strengths. First, the prospective cohort study and MR study have distinct strengths and limitations, enabling them to complement each other to some extent. Second, the consistency between the results of these two studies, which were conducted on Asian and European populations, further strengthens the reliability of the conclusions. Third, the design of the prospective cohort study, combined with the use of LME models to analyze repeated-measures of baPWV, enabled a comprehensive and robust examination of the progression of arterial stiffness.

Nevertheless, it is important to acknowledge the limitations inherent in this study. First, the cohort study was conducted in a single center, and screening for morning serum cortisol may have led to selection bias. Second, potential ethnic variations in cortisol levels and differences in laboratory assay methodologies between the cohort and MR studies must be considered. Third, while our single-timepoint morning cortisol measurement provides a practical assessment for large-scale studies, it primarily reflects the cortisol awakening response and may be susceptible to acute influences such as daily stressors. Although we controlled for multiple known confounders, the lack of psychological data represents a potential limitation, as these unmeasured factors could contribute to residual variability in our findings. Future investigations incorporating multi-timepoint salivary cortisol or 24-hour urinary cortisol measurements, along with standardized psychological assessments, would help validate these relationships. Furthermore, the average follow-up period of 3.5 years of the cohort study may not have been sufficiently long to fully elucidate the relationship between morning cortisol and arterial stiffness.

In conclusion, observational and genetic analyses consistently link higher morning cortisol levels to arterial stiffness, with SBP mediating this relationship. These findings provide novel insights into cortisol’s role in arterial stiffness and potential therapeutic targets.

## Data Availability

The raw data supporting the conclusions of this article will be made available by the authors, without undue reservation.

## References

[1] Kivimäki M Steptoe A . Effects of stress on the development and progression of cardiovascular disease. Nat Rev Cardiol. (2018) 15:215–29. doi: 10.1038/nrcardio.2017.189, PMID: 29213140

[2] Cohen BE Edmondson D Kronish IM . State of the art review: depression, stress, anxiety, and cardiovascular disease. Am J Hypertens. (2015) 28:1295–302. doi: 10.1093/ajh/hpv047, PMID: 25911639 PMC4612342

[3] Vaccarino V Bremner JD . Stress and cardiovascular disease: an update. Nat Rev Cardiol. (2024) 21:603–16. doi: 10.1038/s41569-024-01024-y, PMID: 38698183 PMC11872152

[4] Van den Bergh G Opdebeeck B D’Haese PC Verhulst A . The vicious cycle of arterial stiffness and arterial media calcification. Trends Mol Med. (2019) 25:1133–46. doi: 10.1016/j.molmed.2019.08.006, PMID: 31522956

[5] Cohen S Janicki-Deverts D Miller GE . Psychological stress and disease. JAMA. (2007) 298:1685–7. doi: 10.1001/jama.298.14.1685, PMID: 17925521

[6] Ortiz R Kluwe B Lazarus S Teruel MN Joseph JJ . Cortisol and cardiometabolic disease: a target for advancing health equity. Trends Endocrinol Metab. (2022) 33:786–97. doi: 10.1016/j.tem.2022.08.002, PMID: 36266164 PMC9676046

[7] Joseph JJ Golden SH . Cortisol dysregulation: the bidirectional link between stress, depression, and type 2 diabetes mellitus. Ann N Y Acad Sci. (2017) 1391:20–34. doi: 10.1111/nyas.13217, PMID: 27750377 PMC5334212

[8] Pageau LM Ng TJ Ling J Given BA Robbins LB Deka P . Associations between hair cortisol and blood pressure: a systematic review and meta-analysis. J Hypertens. (2023) 41:875–87. doi: 10.1097/HJH.0000000000003412, PMID: 37016924

[9] Martens A Duran B Vanbesien J Verheyden S Rutteman B Staels W . Clinical and biological correlates of morning serum cortisol in children and adolescents with overweight and obesity. PloS One. (2021) 16:e0258653. doi: 10.1371/journal.pone.0258653, PMID: 34669746 PMC8528324

[10] Mj W Cm T-C Mi G . Association between the metabolic syndrome and serum cortisol in overweight Latino youth. J Clin Endocrinol Metab. (2008) 93:1372–8. doi: 10.1210/jc.2007-2309, PMID: 18252788 PMC2291493

[11] Cohen S Janicki-Deverts D Doyle WJ Miller GE Frank E Rabin BS . Chronic stress, glucocorticoid receptor resistance, inflammation, and disease risk. Proc Natl Acad Sci U.S.A. (2012) 109:5995–9. doi: 10.1073/pnas.1118355109, PMID: 22474371 PMC3341031

[12] Manenschijn L Schaap L van Schoor NM van der Pas S Peeters GMEE Lips P . High long-term cortisol levels, measured in scalp hair, are associated with a history of cardiovascular disease. J Clin Endocrinol Metab. (2013) 98:2078–83. doi: 10.1210/jc.2012-3663, PMID: 23596141

[13] Gump BB Hruska B Heffernan K Brann LS Voss M Labrie-Cleary C . Race, cortisol, and subclinical cardiovascular disease in 9- to 11-year-old children. Health Psychol. (2023) 42:657–67. doi: 10.1037/hea0001300, PMID: 37410422 PMC10601363

[14] Ikeda A Steptoe A Shipley M Abell J Kumari M Tanigawa T . Diurnal pattern of salivary cortisol and progression of aortic stiffness: Longitudinal study. Psychoneuroendocrinology. (2021) 133:105372. doi: 10.1016/j.psyneuen.2021.105372, PMID: 34517196 PMC8543075

[15] Reynolds RM Ilyas B Price JF Fowkes FGR Newby DE Webb DJ . Circulating plasma cortisol concentrations are not associated with coronary artery disease or peripheral vascular disease. QJM. (2009) 102:469–75. doi: 10.1093/qjmed/hcp057, PMID: 19458201

[16] Kumari M Shipley M Stafford M Kivimaki M . Association of diurnal patterns in salivary cortisol with all-cause and cardiovascular mortality: findings from the Whitehall II study. J Clin Endocrinol Metab. (2011) 96:1478–85. doi: 10.1210/jc.2010-2137, PMID: 21346074 PMC3085201

[17] Ebrahim S Davey Smith G . Mendelian randomization: can genetic epidemiology help redress the failures of observational epidemiology? Hum Genet. (2008) 123:15–33. doi: 10.1007/s00439-007-0448-6, PMID: 18038153

[18] Carter AR Sanderson E Hammerton G Richmond RC Davey Smith G Heron J . Mendelian randomisation for mediation analysis: current methods and challenges for implementation. Eur J Epidemiol. (2021) 36:465–78. doi: 10.1007/s10654-021-00757-1, PMID: 33961203 PMC8159796

[19] Ning G . Medical education in diabetes management on the new horizon: insights from metabolic management center. J Diabetes. (2025) 17:e70075. doi: 10.1111/1753-0407.70075, PMID: 40107961 PMC11922674

[20] Zhang Y Wang Y Ning G He P Wang W . Protecting older people: a high priority during the COVID-19 pandemic. Lancet. (2022) 400:729–30. doi: 10.1016/S0140-6736(22)01530-6, PMID: 36058217 PMC9436364

[21] Zhang Y Wang W Ning G . Metabolic Management Center: An innovation project for the management of metabolic diseases and complications in China. J Diabetes. (2019) 11:11–3. doi: 10.1111/1753-0407.12847, PMID: 30284373

[22] Fang Q Shi J Zhang J Peng Y Liu C Wei X . Visit-to-visit HbA1c variability is associated with aortic stiffness progression in participants with type 2 diabetes. Cardiovasc Diabetol. (2023) 22:167. doi: 10.1186/s12933-023-01884-7, PMID: 37415203 PMC10324236

[23] Raz I . MMC celebrating 6 years of experience and expansion. J Diabetes. (2022) 14:356–7. doi: 10.1111/1753-0407.13270, PMID: 35545818 PMC9366589

[24] Liu J Bloomgarden Z . The chinese metabolic management centers. J Diabetes. (2022) 14:362–4. doi: 10.1111/1753-0407.13290, PMID: 35712984 PMC9366566

[25] Whelton PK Carey RM Aronow WS Casey DE Collins KJ Dennison Himmelfarb C . 2017 ACC/AHA/AAPA/ABC/ACPM/AGS/APhA/ASH/ASPC/NMA/PCNA guideline for the prevention, detection, evaluation, and management of high blood pressure in adults: A report of the american college of cardiology/american heart association task force on clinical practice guidelines. J Am Coll Cardiol. (2018) 71:e127–248. doi: 10.1016/j.jacc.2017.11.006, PMID: 29146535

[26] Zhao L Zhou X Chen Y Dong Q Zheng Q Wang Y . Association of visceral fat area or BMI with arterial stiffness in ideal cardiovascular health metrics among T2DM patients. J Diabetes. (2024) 16:e13463. doi: 10.1111/1753-0407.13463, PMID: 37680102 PMC10809303

[27] Wang S Shi J Peng Y Fang Q Mu Q Gu W . Stronger association of triglyceride glucose index than the HOMA-IR with arterial stiffness in patients with type 2 diabetes: a real-world single-centre study. Cardiovasc Diabetol. (2021) 20:82. doi: 10.1186/s12933-021-01274-x, PMID: 33888131 PMC8063289

[28] Bolton JL Hayward C Direk N Lewis JG Hammond GL Hill LA . Genome wide association identifies common variants at the SERPINA6/SERPINA1 locus influencing plasma cortisol and corticosteroid binding globulin. PloS Genet. (2014) 10:e1004474. doi: 10.1371/journal.pgen.1004474, PMID: 25010111 PMC4091794

[29] Larsson SC Lee W-H Burgess S Allara E . Plasma cortisol and risk of atrial fibrillation: A mendelian randomization study. J Clin Endocrinol Metab. (2021) 106:e2521–6. doi: 10.1210/clinem/dgab219, PMID: 33822969 PMC8208666

[30] Crawford AA Soderberg S Kirschbaum C Murphy L Eliasson M Ebrahim S . Morning plasma cortisol as a cardiovascular risk factor: findings from prospective cohort and Mendelian randomization studies. Eur J Endocrinol. (2019) 181:429–38. doi: 10.1530/EJE-19-0161, PMID: 31325907 PMC6733337

[31] Katsuhara S Yokomoto-Umakoshi M Umakoshi H Matsuda Y Iwahashi N Kaneko H . Impact of cortisol on reduction in muscle strength and mass: A mendelian randomization study. J Clin Endocrinol Metab. (2022) 107:e1477–87. doi: 10.1210/clinem/dgab862, PMID: 34850018

[32] Verbanck M Chen C-Y Neale B Do R . Detection of widespread horizontal pleiotropy in causal relationships inferred from Mendelian randomization between complex traits and diseases. Nat Genet. (2018) 50:693–8. doi: 10.1038/s41588-018-0099-7, PMID: 29686387 PMC6083837

[33] Li H Cai J Chen R Zhao Z Ying Z Wang L . Particulate matter exposure and stress hormone levels: A randomized, double-blind, crossover trial of air purification. Circulation. (2017) 136:618–27. doi: 10.1161/CIRCULATIONAHA.116.026796, PMID: 28808144

[34] Toledo-Corral CM Alderete TL Herting MM Habre R Peterson AK Lurmann F . Ambient air pollutants are associated with morning serum cortisol in overweight and obese Latino youth in Los Angeles. Environ Health. (2021) 20:39. doi: 10.1186/s12940-021-00713-2, PMID: 33832509 PMC8034084

[35] Kluwe B Zhao S Kline D Ortiz R Brock G Echouffo-Tcheugui JB . Adiposity measures and morning serum cortisol in african americans: jackson heart study. Obes (Silver Spring). (2021) 29:418–27. doi: 10.1002/oby.23056, PMID: 33491313 PMC9017492

[36] Osborne MT Shin LM Mehta NN Pitman RK Fayad ZA Tawakol A . Disentangling the links between psychosocial stress and cardiovascular disease. Circ Cardiovasc Imaging. (2020) 13:e010931. doi: 10.1161/CIRCIMAGING.120.010931, PMID: 32791843 PMC7430065

[37] Hackett RA Steptoe A . Type 2 diabetes mellitus and psychological stress - a modifiable risk factor. Nat Rev Endocrinol. (2017) 13:547–60. doi: 10.1038/nrendo.2017.64, PMID: 28664919

[38] Joseph JJ Golden SH . Cortisol dysregulation: the bidirectional link between stress, depression, and type 2 diabetes mellitus. Ann N Y Acad Sci. (2017) 1391:20–34. doi: 10.1111/nyas.13217, PMID: 27750377 PMC5334212

[39] Levine GN Cohen BE Commodore-Mensah Y Fleury J Huffman JC Khalid U . Psychological health, well-being, and the mind-heart-body connection: A scientific statement from the american heart association. Circulation. (2021) 143:e763–83. doi: 10.1161/CIR.0000000000000947, PMID: 33486973

[40] Wang Q Shelton RC Dwivedi Y . Interaction between early-life stress and FKBP5 gene variants in major depressive disorder and post-traumatic stress disorder: A systematic review and meta-analysis. J Affect Disord. (2018) 225:422–8. doi: 10.1016/j.jad.2017.08.066, PMID: 28850857 PMC5626653

[41] Tyrka AR Ridout KK Parade SH Paquette A Marsit CJ Seifer R . Childhood maltreatment and methylation of FK506 binding protein 5 gene (FKBP5). Dev Psychopathol. (2015) 27:1637–45. doi: 10.1017/S0954579415000991, PMID: 26535949 PMC4870720

[42] Zannas AS Jia M Hafner K Baumert J Wiechmann T Pape JC . Epigenetic upregulation of FKBP5 by aging and stress contributes to NF-κB-driven inflammation and cardiovascular risk. Proc Natl Acad Sci U.S.A. (2019) 116:11370–9. doi: 10.1073/pnas.1816847116, PMID: 31113877 PMC6561294

[43] Ortiz R Joseph JJ Lee R Wand GS Golden SH . Type 2 diabetes and cardiometabolic risk may be associated with increase in DNA methylation of FKBP5. Clin Epigenet. (2018) 10:82. doi: 10.1186/s13148-018-0513-0, PMID: 29951131 PMC6010037

[44] Nakao M Nomura K Karita K Nishikitani M Yano E . Relationship between brachial-ankle pulse wave velocity and heart rate variability in young Japanese men. Hypertens Res. (2004) 27:925–31. doi: 10.1291/hypres.27.925, PMID: 15894832

[45] Himeno A Satoh-Asahara N Usui T Wada H Tochiya M Kono S . Salivary cortisol levels are associated with outcomes of weight reduction therapy in obese Japanese patients. Metabolism. (2012) 61:255–61. doi: 10.1016/j.metabol.2011.06.023, PMID: 21871641

[46] Violanti JM Burchfiel CM Fekedulegn D Andrew ME Dorn J Hartley TA . Cortisol patterns and brachial artery reactivity in a high stress environment. Psychiatry Res. (2009) 169:75–81. doi: 10.1016/j.psychres.2008.06.012, PMID: 19616310

[47] Arnett DK Blumenthal RS Albert MA Buroker AB Goldberger ZD Hahn EJ . 2019 ACC/AHA guideline on the primary prevention of cardiovascular disease: executive summary: A report of the american college of cardiology/american heart association task force on clinical practice guidelines. Circulation. (2019) 140:e563–95. doi: 10.1161/CIR.0000000000000677 PMC8351755 30879339

[48] Sarafidis PA Bakris GL . Early patterns of blood pressure change and future coronary atherosclerosis. JAMA. (2014) 311:471–2. doi: 10.1001/jama.2013.285123, PMID: 24496535

[49] Crea F . Expanding knowledge in atrial fibrillation, blood pressure treatment, and management of coronary and peripheral artery disease. Eur Heart J. (2024) 45:2795–9. doi: 10.1093/eurheartj/ehae503, PMID: 39150995

[50] Rothwell PM Howard SC Dolan E O’Brien E Dobson JE Dahlöf B . Prognostic significance of visit-to-visit variability, maximum systolic blood pressure, and episodic hypertension. Lancet. (2010) 375:895–905. doi: 10.1016/S0140-6736(10)60308-X, PMID: 20226988

[51] Clark D Nicholls SJ St John J Elshazly MB Ahmed HM Khraishah H . Visit-to-visit blood pressure variability, coronary atheroma progression, and clinical outcomes. JAMA Cardiol. (2019) 4:437–43. doi: 10.1001/jamacardio.2019.0751, PMID: 30969323 PMC6537804

[52] Fan K Wei D Liu X He Y Tian H Tu R . Negative associations of morning serum cortisol levels with obesity: the Henan rural cohort study. J Endocrinol Invest. (2021) 44:2581–92. doi: 10.1007/s40618-021-01558-9, PMID: 33829394

[53] Ishida A Taira H Shinzato T Ohya Y . Association between visceral fat mass and arterial stiffness among community-based screening participants. Hypertens Res. (2023) 46:2488–96. doi: 10.1038/s41440-023-01350-7, PMID: 37353686

[54] Hu L Zhang Y Huang X Song Y Qin X Wang B . Associations between blood pressure indices and brachial-ankle pulse wave velocity in treated hypertensive adults: results from the China stroke primary prevention trial (CSPPT). Sci Rep. (2019) 9:8178. doi: 10.1038/s41598-019-44740-z, PMID: 31160658 PMC6547652

[55] Deng X Song Y Han X Chen X Yang W Wu S . Brachial-ankle pulse wave velocity trajectories in a middle-aged population. Front Cardiovasc Med. (2023) 10:1092525. doi: 10.3389/fcvm.2023.1092525, PMID: 37051065 PMC10083284

[56] Huang W Gan Z Gao Z Lin Q Li X Xie W . Discrepancies between general and central obesity in arterial stiffness: observational studies and Mendelian randomization study. BMC Med. (2024) 22:325. doi: 10.1186/s12916-024-03546-1, PMID: 39113079 PMC11304581

