# Supplementary material for: Morning cortisol as an indicator of arterial stiffness in patients with type 2 diabetes: prospective cohort study and Mendelian randomization study
Source: Front Endocrinol (Lausanne). 2025 Nov 25;16:1687909. doi: 10.3389/fendo.2025.1687909 (PMC12685639; doi:10.3389/fendo.2025.1687909)
Supplement: Supplementary file 1 [file DataSheet1.docx]

Supplementary Material

# Supplementary Tables and Figures

## Supplementary Tables

**Supplementary Table S1.** Genetic instruments of morning cortisol

| **Gene** | **SNP** | **Effect allele** | **Other allele** | **EAF** | **β** | **SE** | ***P* value** | **N** | **F-statistics** |
| --- | --- | --- | --- | --- | --- | --- | --- | --- | --- |
| *SERPINA6* | rs11621961 | T | C | 0.356845 | -0.08 | 0.014 | 3.97E-08 | 11438 | 30.21 |
| *SERPINA6* | rs12589136 | T | G | 0.216641 | 0.10 | 0.014 | 3.31971E-12 | 12589 | 48.56 |
| *SERPINA1* | rs2749527 | T | C | 0.488791 | -0.08 | 0.01 | 5.20955E-11 | 12589 | 43.17 |

*SERPINA1*, serpin family A member 1; *SERPINA6*, serpin family A member 6; SNP, single-nucleotide polymorphism; SE, standard error; eaf, effect allele frequency.

**Supplementary Table S2.** Overview of the source of GWAS data

| **GWAS ID** | **Year** | **Trait** | **Consortium** | **Sample size** | **Number**  **of SNPs** | **Population** |
| --- | --- | --- | --- | --- | --- | --- |
| ieu-b-39 | 2018 | Diastolic blood pressure | International Consortium of Blood Pressure | 757,601 | 7,160,619 | European |
| ieu-b-38 | 2017 | Systolic blood pressure | International Consortium of Blood Pressure | 757,601 | 7,088,083 | European |
| ukb-d-I9_CORATHER | 2018 | Coronary atherosclerosis | NA | 361,194 | 13,586,589 | European |
| ieu-a-1012 | 2014 | Morning cortisol | CORNET | 12,597 | 2,660,192 | European |

**Supplementary Table S3.** Linear mixed-effects models mediation analysis of morning serum log_10_Cortisol on arterial stiffness via blood lipids, blood glucose and lifestyles.

|  | **Risk factors** | **Step 1 (β1)** | **Step 2 (β2)** | **Indirect Effect (β1×β2）** |
| --- | --- | --- | --- | --- |
| Blood lipids | TC | -0.04 (-0.18, 0.09) | **/** | **/** |
|  | TG | 0.18 (-0.08, 0.44) | **/** | **/** |
|  | HDL-C | 0.00 (-0.03, 0.04) | **/** | **/** |
|  | LDL-C | -0.09 (-0.22, 0.04) | **/** | **/** |
| Blood glucose | FBG | 1.04 (0.60, 1.48) *** | 0.01 (-0.02, 0.04) | / |
| Lifestyles | Smoking | -0.07 (-0.16, 0.02) | **/** | **/** |
|  | Drinking | 0.02 (-0.02, 0.07) | **/** | **/** |
|  | Sleep duration | 0.04 (-0.05, 0.13) | **/** | **/** |

*P < 0.05, **P < 0.01, ***P < 0.001.

Log_10_, log-transformed with base 10; TC, total cholesterol; TG, triglycerides; HDL-C, high density lipoprotein cholesterol; LDL-C, low density lipoprotein cholesterol; FBG, fasting blood glucose. Smoking (current, former and quit≤ 12 months, never or quit>12 months), drinking (yes or no), sleep duration (< 7 h, 7-9 h, > 9 h).

β1, the effect of morning serum log10Cortisol on risk factor. β2, the effect of risk factors on arterial stiffness. P values were calculated from the restricted maximum likelihood. Log10, log-transformed with base 10.

**Supplementary Table S4.** Sensitivity analysis models in the cohort study.

| **Model** | **Covariables** |
| --- | --- |
| 1 (Main model) | Age, sex, MAP, diabetes duration, VFA, LDL-C, HbA1c, smoking status, drinking status, history of cardiovascular disease, antihypertensive agents and lipid-lowering agents |
| 2 | Age, sex, MAP, diabetes duration, **-VFA**, LDL-C, HbA1c, smoking status, drinking status, history of cardiovascular disease, antihypertensive agents and lipid-lowering agents |
| 3 | Age, sex, MAP, diabetes duration, VFA, **-LDL-C**, HbA1c, smoking status, drinking status, history of cardiovascular disease, antihypertensive agents and lipid-lowering agents |
| 4 | Age, sex, MAP, diabetes duration, VFA, LDL-C, **-HbA1c**, smoking status, drinking status, history of cardiovascular disease, antihypertensive agents and lipid-lowering agents |
| 5 | Age, sex, MAP, diabetes duration, VFA, LDL-C, HbA1c, smoking status, drinking status, history of cardiovascular disease, **-antihypertensive agents and lipid-lowering agents** |

MAP, mean arterial pressure; VFA, visceral fat area; LDL-C, low-density lipoprotein cholesterol; HbA1c, glycated hemoglobin A1c.

**Supplementary Table S5.** Sensitivity analysis results in the cohort study.

|  | **β （95%CI)** | ***P* value** |
| --- | --- | --- |
| Model 2 | 0.54 (0.13, 0.94) | **0.009** |
| Model 3 | 0.68 (0.25, 1.10) | **0.002** |
| Model 4 | 0.71 (0.29, 1.13) | **0.001** |
| Model 5 | 0.67 (0.25, 1.09) | **0.002** |

**Supplementary Table S6.** Detailed results of sensitivity analyses for two-step MR study

| Exposure | Outcome | *P_IVW_* | *P_MR-Egger intercept_* | *P_IVW.Q_* |
| --- | --- | --- | --- | --- |
| Morning cortisol | Coronary atherosclerosis | 0.049 | 0.603 | 0.530 |
| Morning cortisol | Systolic blood pressure | 0.007 | 0.640 | 0.720 |
| Morning cortisol | Diastolic blood pressure | 0.069 | 0.443 | 0.489 |
| Diastolic blood pressure | Coronary atherosclerosis | 2.354E-138 | 0.090 | 6.458E-170 |
| Systolic blood pressure | Coronary atherosclerosis | 2.341E-116 | 0.667 | 3.439E-181 |

## Supplementary Figures


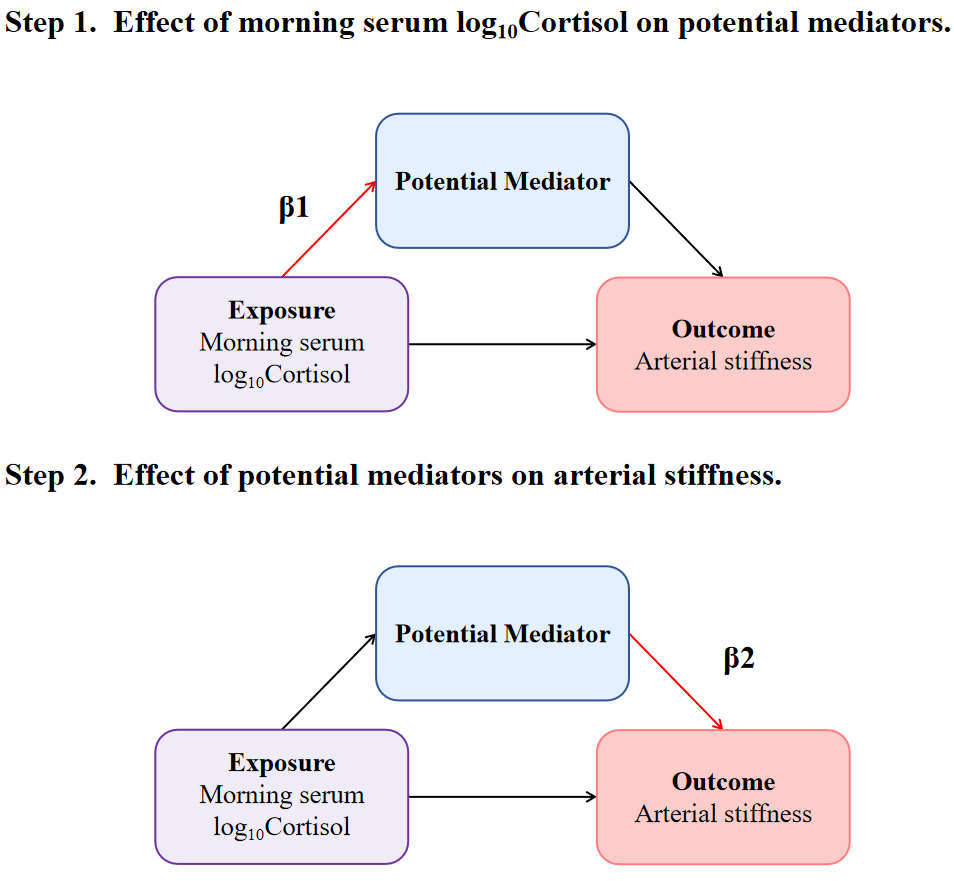


**Supplementary Figure S1.** Mediation analysis of morning serum cortisol on arterial stiffness via potential mediators.


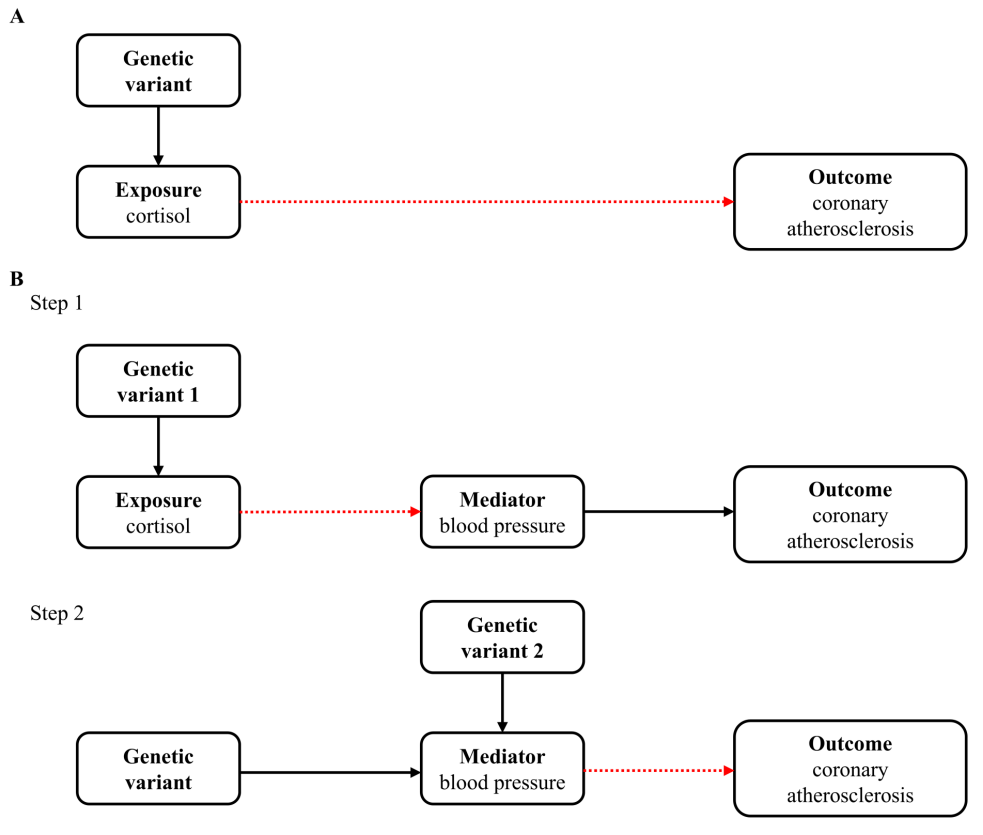


**Supplementary Figure S2.** Schematic diagram of MR study.

**A**, standard Mendelian randomization (MR) analysis of cortisol; **B**, two-step MR analysis of mediation by blood pressure. Two-step MR tests the association between a genetic variant and the exposure (cortisol) postulated to influence the outcome (coronary atherosclerosis) via an altered mediator (blood pressure). Broken arrows indicate the causal pathway to be assessed.


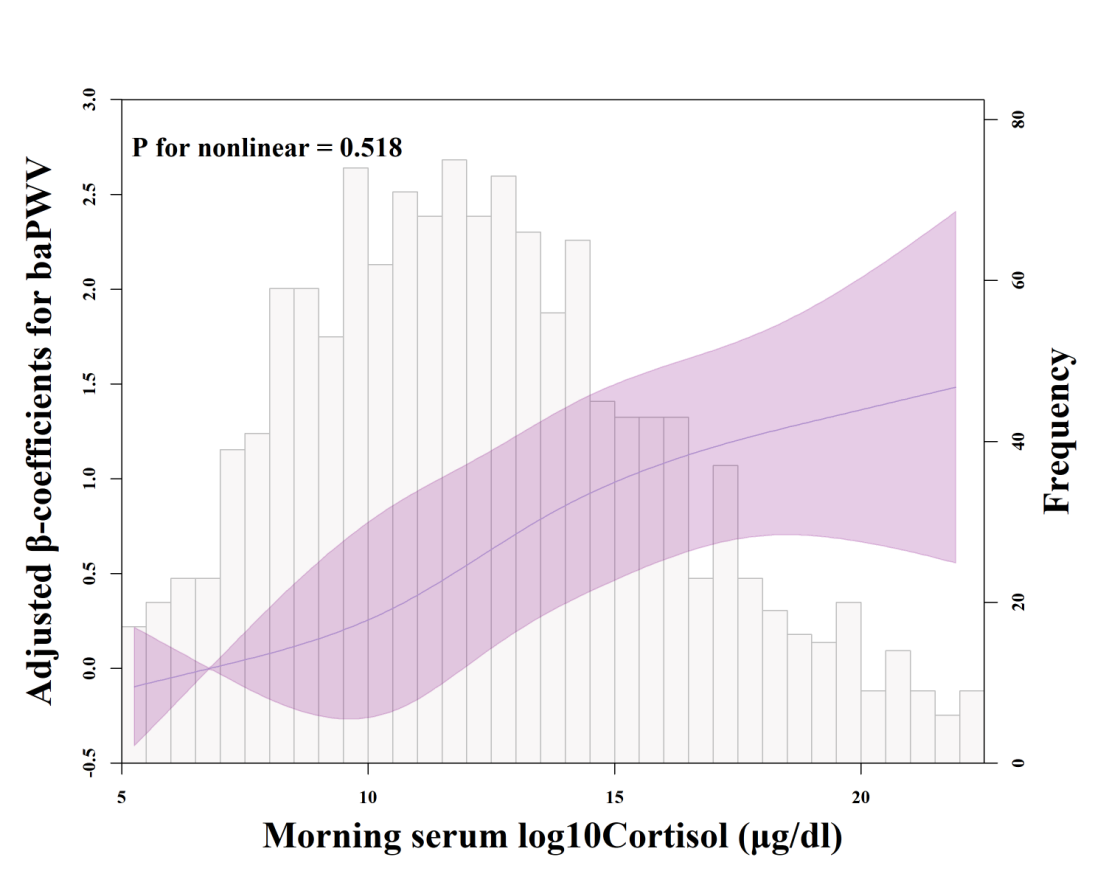


**Supplementary Figure S3.** RCS plot for dose–response relationship between baseline morning serum cortisol and follow-up baPWV.

Linear mixed-effects model adjusted for Model 2: age, sex, MAP, diabetes duration, VFA, LDL-C, HbA1c, smoking status, drinking status, history of CVD, antihypertensive agents, and lipid-lowering agents.

RCS, restricted cubic spline; baPWV, brachial-ankle pulse wave velocity; log10, log-transformed with base 10; MAP, mean arterial pressure; VFA, visceral fat area; LDL-C, low-density lipoprotein cholesterol; HbA1c, glycated hemoglobin A1c; CVD, cardiovascular disease.
